# Factors associated with adolescent cigarette smoking in Greece: Results from a cross sectional study (GYTS Study)

**DOI:** 10.1186/1471-2458-8-313

**Published:** 2008-09-15

**Authors:** George Rachiotis, Adamson S Muula, Emmanuel Rudatsikira, Seter Siziya, Athina Kyrlesi, Konstantinos Gourgoulianis, Christos Hadjichristodoulou

**Affiliations:** 1 Department of Hygiene and Epidemiology, School of Medicine, University of Thessaly, Greece; 2 Department of Community Health, University of Malawi, College of Medicine, Blantyre, Malawi; 3 Departments of Epidemiology and Biostatistics and Global Health, Loma Linda University, School of Public Health, Loma Linda, California, USA; 4 Department of Community Medicine, University of Zambia, Lusaka, Zambia; 5 Department of Pulmonology, School of Medicine, University of Thessaly, Greece

## Abstract

**Background:**

Data about the predictors of smoking among adolescents in Greece are sparse. We tried to identify factors associated with current cigarette smoking among in-school adolescents in Greece in the context of GYTS study.

**Methods:**

A secondary analysis of data from a questionnaire study using the Global Youth Tobacco Survey methodology was conducted to identify factors associated with smoking among adolescents in Greece. Data were collected in 2004–2005. The outcome variable was cigarette smoking within the past 30 days preceding the survey while independent variables included age, gender, parental educational status, parental smoking, perception of harmfulness of smoking, and the amount of pocket money at the adolescent's disposal.

**Results:**

6141 adolescents (51.5% males and 48.5% females) participated in the study. In multivariate analysis, cigarette smoking was associated with male gender (OR: 1.62; 95% CI: 1, 08–3.08), parental smoking (OR: 2.59; 95% CI: 1.45–5.89), and having pocket money ≥ 16 Euros (OR: 2.64; 95% CI: 1.19–5.98).

**Conclusion:**

Male gender, parental smoking, and having pocket-money ≥ 16 Euros were independently associated with current smoking among Greek students. These findings could be taken into account in order to formulate a comprehensive anti-smoking strategy in Greece.

## Background

Tobacco is a leading cause of illness in the developed world where non-communicable diseases contribute to the greatest burden of disease [1]. Many adult smokers initiate the smoking habit during adolescence or as young adults [2]. Much of the severe health consequences of smoking are in adulthood. However, there are also significant short and intermediate term effects of smoking that may be experienced such as asthma and chronic cough [3,4]. Information on adolescent smoking is valuable not just because smoking is independently associated with adverse health effects, but also through the clustering of other harmful lifestyles among adolescents who smoke [5]. Arvanitidou et al have reported that the odds of alcohol use among adolescents in Greece who reported smoking habit were 5.2 compared to non-smokers [6].

Known risk factors associated with cigarette smoking among adolescents include age [7], gender [7,8], having smoking friends and or smoking parents, the perception that smoking is or not harmful [9], and the amount of pocket money [10].

Greece is a leading tobacco-producer country within European Union. In addition, in Greece the prevalence of smoking is 37% and the annual per capita consumption of cigarettes was one of the highest in the European Union [11,12].

Kyrlesi et al have reported on the prevalence of tobacco use among middle school adolescents in Greece using the same data from which our study is based [13]. The study by Kyrlesi et al only reported on prevalence of tobacco use, exposure to second hand smoke, tobacco related media and advertising, cessation experience and access of tobacco products. Factors associated with cigarette smoking were not explored. Using the same database, we explored the factors that may be associated with cigarette smoking among in-school adolescents in Greece. We believe while knowledge of prevalence of various behaviors is important, understanding the factors that may be associated with the behavior may better inform public health policy decision.

## Methods

Our study involved the secondary analysis of the Global Youth Tobacco Survey (GYTS) conducted in Greece among middle-school students in Greece, 2004–2005. A comprehensive description of the data collection methodology was reported previously by Kyrelsi et al [13]. In brief, a two-stage cluster sampling design was instituted in which in the first phase all schools containing the middle-school grades in Greece were identified and 100 schools were selected (25 schools from each region). This was considered adequate to obtain a sample design that would produce representative estimates for each region. In the first stage of sampling, the probability of schools being selected was proportional to the number of students enrolled in the specified grades (grades 1–3 at all middle schools). In the second sampling stage, classes within the selected schools were randomly selected. All students in selected classes attending school on the day of the survey were eligible to participate. The Committee on Health Promotion of the Ministry of National Education and Religions approved the survey. Parents were notified by a letter in advance of the survey and students gave verbal consent to complete the questionnaire. Further description of the GYTS methodology has been described elsewhere [14].

### Data collection

The GYTS questionnaire included data demographic variables and experience with cigarette smoking. Self-completed questionnaires were used. A project coordinator supervised the data collection process and reported to supervisor on a daily basis. Completed questionnaires were sent to the Centers for Disease Control and Prevention for processing where they were transformed into electronic files.

### Statistical analyses

A weighting factor was used in the analysis to reflect the likelihood of sampling each student and to reduce bias by compensating for differing patterns of non response. The weight used for estimation is given by the following formula:

W = W1 * W2 * f1 * f2 *f3 *f4, where

W1 = the inverse of the probability of selecting the school

W2 = the inverse of the probability of selecting the classroom within the school

fl = a school-level non response adjustment factor calculated by school size category (small, medium, large)

f2 = a class-level non response adjustment factor calculated for each school

f3 = a student-level non response adjustment factor calculated by class

f4 = a post stratification adjustment factor calculated by grade.

We conducted logistic regression analysis using SUDAAN software version 9.0 (Research Triangle Institute, NC, USA) to estimate the association between relevant predictor variables and current cigarette. To assess current smoking status participants were asked "During the past 30 days, on how many days did you smoke cigarettes?" In the analysis current smokers were those who reported having smoked one or more days during the last 30 days preceding the survey. The study was based on adolescents who by some authors are defined as 12 to 18 years; however age of adolescence varies by culture and much younger and much older persons may be included in the definition. In the Greece GYTS, the data collection questionnaire asked the question:

How old are you? The options available were: a) 11 years or young; b) 12 years: c) 13 years; d) 14 years; d) 15 years; e) 16 years and f) 17 years or older.

We report unadjusted Odds Ratios for selected predictor variables while considering current cigarette smoking as dependent variable. We thereafter report results of adjusted odds ratios for the factors.

## Results

Table 1 presents the characteristics of the population under study.

**Table 1:** Characteristics of the Study Population

	Total N (%)	Males N (%)	Females N (%)
**Age (years)**	6146 (100)	3096 (50.4)	3045 (49.6)
11–12	627 (10.2)	357 (11.5)	270 (8.9)
13	1757 (28.6)	884 (28.6)	873 (28.6)
14	1910 (31.1)	911 (29.4)	999 (32.8)
15	1465 (23.9)	730 (23.6)	735 (24.1)
16–17	382 (6.2)	214 (6.9)	168 (5.6)
**Education father/stepfather**			
Elementary school or less	1296 (19.8)	663 (21.4)	477 (15.7)
Middle school	1025 (16.1)	493 (15.9)	502 (16.5)
High school	1803 (28.3)	834 (27.0)	934 (30.7)
University	2238 (36.4)	1106 (35.7)	1132 (37.1)
**Education mother/stepmother**			
Elementary school or less	950 (15.5)	521 (16.8)	429 (14.1)
Middle school	911 (14.8)	433 (14.0)	478 (15.7)
High school	2006 (32.7)	990 (32.0)	1016 (33.3)
University	2274 (37.0)	1152 (37.2)	1122 (36.9)
**Visiting grand parents**			
Every day	2266 (37.3)	1199 (39.1)	1067 (35.4)
2–5 times/week	2153 (35.4)	1080 (35.2)	1073 (35.7)
Once a month or less	429 (7.1)	202 (6.6)	227 (7.5)
Never	1231 (20.2)	585 (19.1)	646 (21.4)
**Parental smoking**			
None	1947 (32.1)	1020 (33.3)	927 (30.8)
Father only	1589 (26.2)	819 (26.8)	770 (25.6)
Mother only	724 (11.9)	347 (11.3)	377 (12.5)
Both parents	1813 (29.9)	873 (28.5)	940 (31.2)
**Smoking status grand parents**			
None	3146 (53.4)	1568 (52.8)	1578 (54.0)
One or more grand parents	2747 (46.6)	1404 (47.2)	1343 (46.0)
**Accepting cigarette offered by one of best one's friends smoking**			
Definitely not	4287 (70.7)	2166 (70.7)	2121 (70.7)
Probably not	873 (14.4)	441 (14.4)	432 (14.4)
Probably yes	697 (11.5)	342 (11.2)	355 (11.8)
Definitely yes	204 (3.4)	111 (3.6)	93 (3.1)
**Perception cigarettes smoking harmful**			
Definitely yes or probably yes	5573 (91.7)	2760 (90.1)	2813 (93.2)
Definitely not or probably not	507 (8.3)	303 (9.9)	204 (6.8)
**Pocket money**			
< 7 Euro	2267 (44.7)	1371 (45.6)	1296 (43.7)
8–15 Euro	1647 (27.6)	753 (25.1)	894 (30.1)
16–23 Euro	709 (11.9)	348 (11.6)	361 (12.2)
24–31 Euro	369 (6.2)	202 (6.7)	167 (5.6)
>=32 Euro	581 (9.6)	332 (11.0)	249 (8.4)
**Current cigarette smoking**			
No	5174 (87.5)	2576 (86.7)	2597 (88.2)
Yes	741 (12.5)	394 (13.3)	347 (11.8)

From 7126 eligible students 6378 actually took part (response rate 89%). Among males at age groups 11–12, and 16–17 years old the prevalence of smoking was 9.4%, and 48.2%, respectively.

Among females at these age groups the prevalence of smoking was 12.8% for age group 11–12 years, and 47.6% for age group 16–17 years.

### Factors associated with smoking in bivariate analysis (table 2)

**Table 2:** Variables associated with current smoking (Bi-variate analysis)

	Total Odds ratio (95% confidence interval)	Males Odds ratio (95% confidence interval)	Females Odds ratio (95% confidence interval)
**Age (years)**			
11–12	1.00	1.00	1.00
13	**0.30 [0.22, 0.41]**	**0.56 [0.35, 0.89]**	**0.20 [0.11, 0.35]**
14	**0.50 [0.31, 0.79]**	**0.63 [0.40, 0.97]**	**0.47 [0.36, 0.63]**
15	**1.05 [0.81, 1.38]**	**1.65 [1.09, 2.51]**	1.04 [0.66, 1.64]
16–17	**5.27 [2.96, 9.39]**	**7.21 [4.42, 11.76**	**4.89 [3.48, 6.86]**
**Gender**			
Females	1.00		
Males	**1.26 [1.05, 1.50]**		
**Education father/stepfather**			
Elementary school or less	1.00	1.00	1.00
Middle school	0.81 [0.56, 1.17]	0.76 [0.58, 1.00]	0.85 [0.55, 1.33]
High school	**0.53 [0.40, 0.69]**	**0.47 [0.33, 0.68]**	**0.60 [0.41, 0.90]**
University	**0.52 [0.42, 0.69]**	**0.54 [0.39, 0.75]**	**0.49 [0.32, 0.77]**
**Education mother/stepmother**			
Elementary school or less	1.00	1.00	1.00
Middle school	1.00 [0.74, 1.34]	0.95 [0.62, 1.46]	0.71 [0.47, 1.08]
High school	**0.54 [0.41, 0.71]**	**0.48 [0.32, 0.71]**	**0.61 [0.41, 0.93]**
University	**0.53 [0.41, 0.71]**	**0.55 [0.38, 0.81]**	**0.61 [0.42, 0.89]**
**Parental smoking**			
None	1.00	1.00	1.00
Father only	**1.57 [1.22, 2.02]**	**1.87 [1.34, 2.61**]	1.27 [0.82, 1.97]
Mother only	1.38 [1.00, 1.89]	1.11 [0.69, 1.81]	**1.67 [1.07, 2.60]**
Both parents	**2.48 [1.99, 3.09]**	**2.72 [1.98, 3.74]**	**2.13 [1.54, 2.96]**
**Perception cigarettes smoking harmful**			
Definitely not or probably not	1.00	1.00	1.00
Definitely yes or probably yes	1.20 [0.92, 1.56]	1.13 [0.88, 1.45]	**1.23 [1.04, 1.47]**
**Pocket money**			
< 7 Euro	1.00	1.00	1.00
8–15 Euro	**2.29 [1.53, 3.41]**	**2.06 [1.23, 3.45]**	**3.14 [1.50, 6.60]**
16–23 Euro	**3.39 [2.34, 4.91]**	**3.31 [2.03, 5.39]**	**4.91 [2.47, 9.75]**
24–31 Euro	**4.15 [2.78, 6.20]**	**4.03 [2.37, 6.85]**	**6.04 [2.92, 12.47]**
>=32 Euro	**7.26 [4.73, 11.14]**	**6.78 [3.86, 11.91]**	**10.96 [5.09, 23.58]**

Males were more likely to smoke than females (OR = 1.26; 95% CI [1.05, 1.50]). Subjects aged 13–14 years were less likely to smoke than those who were 11–12 years old (OR = 0.30; 95% CI [0.22, 0.41] for 13 years old and OR = 0.50; 95% CI [0.81, 1.38] for 14 years old) while the oldest individuals (16–17 years old) were more than 5 times likely to smoke those aged 11–12 years (OR = 5.27; 95% CI [2.96, 9.39]).

For both males and females respondents, having both parents smokers was associated with a more than two times the odds of smoking (OR = 2.72; 95% CI [1.98, 3.74) for males and OR = 2.13; 95% CI [1.54, 2.96] for females). Boys with only father smoking were more likely to smoke than those who had nonsmoking fathers (OR = 1.87; 95% CI [1.34, 2.61]). Likewise, girls with only mother smoking were more likely to smoke than those with nonsmoking mothers (OR = 1.67; 95% CI [1.07, 2.60]).

Subjects whose parents had higher school education or higher were less likely to smoke than those whose parents had elementary education or less. Pocket money was associated with increased odds of smoking. Compared to boys who reported having seven Euro or less per week, those who had more 32 Euro or more were more than six times likely to smoke (OR = 6.78; 95% CI [3.86, 11.91]). For girls, those who had more than 32 Euro per week were more than 10 times likely to smoke than those who had seven Euro or less (OR = 10.96; 95% CI [5.09, 23.58]).

### Variables associated with smoking in multivariate analysis (table 3)

**Table 3:** Variables associated with current smoking in multivariate analysis among adolescents in Greece, 2005

Variable	Odd ratios with 95% CI
**Age (years)**	
11–12	1.00
13	**0.42 [0.23, 0.93]**
14	**0.37 [0.27, 0.87]**
15	0.86 [0.39, 1.82]
16–17	1.75 [0.79, 4.08]
**Gender**	
Females	1.00
Males	**1.62 [1.08, 3.08]**
**Education father/stepfather**	
Elementary school or less	1.00
Middle school	0.78 [0.37, 1.87]
High school	0.67 [0.33, 1.49]
University	0.89 [0.38, 1.74]
**Education mother/stepmother**	
Elementary school or less	1.00
Middle school	0.71 [0.40, 1.89]
High school	1.02 [0.47, 2.32]
University	0.89 [0.34, 2.28]
**Parental smoking**	
None	1.00
Father only	**2.49 [1.12, 6.08]**
Mother only	2.38 [0.91, 5.36]
Both parents	**2.59 [1.45, 5.89]**
**Pocket money**	
< 7 Euro	1.00
8–15 Euro	2.22 [0.98, 5.43]
16–23 Euro	**2.64 [1.19, 5.58]**
24–31 Euro	**3.46 [1.41, 7.79]**
>=32 Euro	**4.93 [1.59, 14.21]**

Males subjects were more likely to smoke than females (OR = 1.62; 95% CI [1.08, 3.08]). Compared to teens who had nonsmoking parents, those whose both parents or whose father only were smokers were more than twice likely to smoke (OR = 2.59; 95% CI [1.45, 5.89] and OR = 2.49; 95% CI [1.12, 6.08] respectively).

Subjects who reported having more 16 Euro per week or more were more likely to smoke than those who had seven Euro or less (OR = 2.64; 95% CI [1.19, 5.58] for 16–23 Euro per week, OR = 3.46; 95% CI [1.41, 7.79] for 24–31 Euro/week, and OR = 4.93; 95% CI [1.59, 14.21 for more than 32 Euro/week). There was a dose-response relationship between smoking and the amount of pocket money respondents spent per week (p-value for trend = 0.03).

## Discussion

Using the GYTS, Kyrlesi et al (2007) [13] reported that among 13 to 15 year olds, 16.2% were current users of all tobacco products, 1 in 4 had started smoking by 10 years and 94.1% reported environmental tobacco exposure at home. 10.4% were current cigarette smoking among 13 to 15 year olds. However, the predictors of smoking or social correlates were not reported among school-going adolescents in Greece. Our study explored the association between a selected list of variables and current cigarette smoking.

In bivariate analysis, we found that older age, male gender, having smoking parents, lower parental education status, and high amount of pocket money at the adolescent's disposal were positively associated with being a current cigarette smoker. In multivariate analysis, male gender, smoking status of parents and pocket money were positively associated with being a current smoker. Previous studies elsewhere have reported the association between cigarette smoking and age, gender, parental smoking status, and pocket money [7-10].

Males were more likely to be smokers than females. Similar findings have been reported in other studies and may suggest societal tolerance of male smoking [9,15,16]. However, Hublet et al.(2006) have reported that female adolescents have higher prevalence of cigarette smoking than males in Sweden (13.7% vs. 5.5%), Norway (19.9% vs. 15.4%), Austria (24.7% vs. 19.5%), Belgium (19.0 vs. 16.8%) and Finland (18.0 vs. 16.4) [8], which is an indication that gender difference in cigarette smoking may be context-specific. Furthermore, it's of considerable interest that in some countries (e.g. Greece, Japan, Malawi and Ethiopia) apparently smoking is more prevalent among adolescents boys than girls, in other countries (e.g. Sweden, Norway, Austria, Finland, NZ, Australia) smoking is more prevalent among adolescent girls. In particular, for Greece we speculate that the previous finding could be associated with the ongoing – and not yet completed – process of ''westernization" of the Greek society.

Findings from this study indicate that subjects whose parents smoked were more likely to be smokers than those whose parents were not smokers; which is consistent with previous studies [9]. This suggests the influence parents have on the lifestyle of their children.

In this study, subjects who had more than 16 Euro per week as pocket money were more likely to smoke than those who had seven Euros or less per week. This finding may suggest that having disposable income may influence smoking practice and/or that those adolescents with no money or very little may be purchasing bare essentials. Mohan et al have also reported on a four times the risk of being a smoker among adolescents who received pocket money versus none in India [10]. The public health significance of this finding is that parents and other guardians who provide adolescents with cash should take interest in how that money is used.

We also found that the higher the educational level of the father, the less likely that the teenager was a smoker. On the other hand, it did not appear that the educational status of the mother was associated with being a smoker or non-smoker. We suggest that it is possible that the educational status, and therefore the socio-economic status of the male parent could influence adolescent behavior more than that of the mother.

Our study has the following limitations. Firstly the data were collected through self-completion of the questionnaire. It is possible that recall bias could affect the accuracy of the reports as well as deliberate miss-reporting. As our assessment of current smoking status was not validated by biomarkers such as nicotine or cotinine levels or exhaled carbon monoxide, it is difficult to estimate the extent of any reporting biases that may have occurred [17,18]. However our study used a standardized questionnaire that enables within country and across country comparisons of smoking status. The prevalence estimates also obtained are likely to closely represent the smoking prevalence among school-going adolescents. It is not known how representative our sample was to out of school adolescents.

As it has been noted above [11], Greece recorded a high smoking prevalence (37–40% of total population are current smokers). It has been suggested that Greece probably presents a pattern of smoking epidemic similar to that observed in United States and Western Europe during the sixties when more than 40% of adult smoked and smoking rates were almost equal between socioeconomic groups [19,20]. In addition a study on the prevalence of current smoking among students (GYTS project) revealed that 16% of the adolescents aged 13–15 years were current smokers [13]. The high prevalence of smoking among adults and adolescents reflects the state of antismoking and public health activities in Greece. Indeed, the first (and the only one till now) nationwide antismoking campaign has been implemented in 1978 [21].

We believe that there is an urgent need for designing and implementing a national programme against smoking in Greece. In the context of that programme special attention should be paying to the determinants of smoking among students.

## Conclusion

Among school-going adolescents in Greece, we found that cigarette smoking is strongly associated with the amount of pocket money as well as the smoking status of parents. These findings indicate the need to implement public health interventions paying attention to the determinants of smoking in this group.

## Competing interests

The authors declare that they have no competing interests.

## Authors' contributions

GR participated in the interpretation of data, drafting and revision of manuscript. ASM participated in the data analysis, interpretation of findings, and drafting of manuscript. ER designed the analysis plan, conducted analysis of data, and participated in the interpretation of data, and drafting of manuscript. SS participated in the interpretation of data, and drafting of the manuscript. AK participated in data collection, interpretations of findings, and drafting of the manuscript. KG has been involved in drafting and revising the manuscript for important intellectual content. CH participated in the development of the methodology of the study, supervised the data collection process, and developed the questionnaire. CH supervised the preparation of the manuscript. All authors have read and approved the manuscript.

## Pre-publication history

The pre-publication history for this paper can be accessed here:


